# Supplementary material for: A high-resolution mRNA expression time course of embryonic development in zebrafish
Source: eLife. 2017 Nov 16;6:e30860. doi: 10.7554/eLife.30860 (PMC5690287; doi:10.7554/eLife.30860)
Supplement: Supplementary file 6. [file elife-30860-supp6.zip › biolayout-clusters-files/Cluster166-genes.html]

Cluster166


# Cluster166: Genes

| Ensembl ID | Gene Name | Chr | Start | End | Biotype |
| --- | --- | --- | --- | --- | --- |
| ENSDARG00000074033 | adamtsl2 | 5 | 65201775 | 65242421 | protein\_coding |
| ENSDARG00000040352 | crot | 16 | 28667351 | 28692945 | protein\_coding |
| ENSDARG00000098746 | dhrs13l1 | 14 | 4044395 | 4054791 | protein\_coding |
| ENSDARG00000021135 | dhrs2 | 7 | 23768307 | 23775873 | protein\_coding |
| ENSDARG00000052271 | slc22a18 | 7 | 39266734 | 39281351 | protein\_coding |
| ENSDARG00000027986 | slc35d1b | 2 | 5882005 | 5898444 | protein\_coding |
| ENSDARG00000055226 | slc7a7 | 7 | 19310077 | 19335574 | protein\_coding |
| ENSDARG00000095464 | zgc:66350 | 5 | 9542005 | 9551703 | protein\_coding |
